# Rectal prolapse: tailoring operative strategy to pathophysiology

**DOI:** 10.1007/s10151-025-03265-6

**Published:** 2026-01-13

**Authors:** M. A. Boom, E. C. J. Consten

**Affiliations:** 1 Department of Surgery, UMC Groningen, Groningen, The Netherlands; 2 Department of Surgery, Meander Medical Centre, Amersfoort, The Netherlands

Surgery for rectal prolapse has long been shaped as much by the heterogeneity of methods and measurements as by true differences between operations. A recent multicenter audit of ventral mesh rectopexy (VMR) demonstrated striking inter-hospital variation in indication, operative steps, fixation, and peri-operative pathways, underlining how inconsistent reporting and follow-up dilute any comparative inference [1]. Against this background, the two recent *Controversies in Coloproctology* papers, one advocating VMR and the other defending resection rectopexy (RR), should be read less as opposing manifestos than as complementary calls for phenotype-driven, standardized surgery [2, 3].

## Evidence across abdominal options

Randomized comparisons consistently show that abdominal procedures outperform perineal repairs in fit patients. The DELORES trial demonstrated a fourfold lower 2-year recurrence after laparoscopic resection rectopexy than after Delorme (8% versus 43%), together with better continence and quality-of-life outcomes [4]. The large PROSPER trial found broadly comparable recurrence across abdominal techniques, although its factorial design was underpowered to detect superiority of one abdominal procedure over another [5]. A more recent multicenter randomized study likewise confirmed similar functional gains, but highlighted that true long-term recurrence remains substantial [6].

National registries data confirm the excellent safety profile of laparoscopic abdominal approaches, with elective mortality around 0.3% and a marked shift toward minimally invasive surgery over the past two decades [7, 8]. Single-center experience mirrors this evolution, with a sustained move toward VMR and away from posterior or sutured rectopexy [9]. It is therefore unsurprising that, in patients fit for an abdominal approach, contemporary European practice has progressively consolidated around laparoscopic techniques and, in particular, VMR, supported by registry data, multicenter audits, and international consensus statements emphasizing its nerve-sparing rationale and the need for standardized technique and follow-up [1, 8–16].

Consistent with this evolution, contemporary systematic reviews help reconcile these disparate data. In pooled studies of internal rectal prolapse, VMR achieved greater improvement in both constipation (~77%) and fecal incontinence (~63%), with no statistically significant difference in recurrence versus RR; RR was associated with longer operating time and a higher rate of major complications [17].

A dedicated meta-analysis comparing mesh-based rectopexy with RR likewise found no significant difference in recurrence rates, complications, or functional outcomes, and trial-sequential analysis underscored that further high-quality comparative studies are needed before declaring any gold standard [18]. A conclusion driven largely by heterogeneity in patient phenotypes, variable follow-up, and non-uniform definitions of recurrence.

## Why a ventral, nerve-sparing logic resonates

For external prolapse or high-grade internal prolapse with incontinence or obstructed defecation but without slow-transit features, VMR offers a mechanistically coherent solution: it avoids posterolateral mobilization, aligns with the anterior-defect logic, and preserves autonomic fibers—features that plausibly underpin its superior functional outcomes [1, 10, 11, 15]. In experienced centers, laparoscopic or robotic VMR integrates seamlessly with enhanced recovery after surgery (ERAS) pathways and with concomitant pelvic organ prolapse repair when multicompartment defects co-exist. Large combined sacrocolpopexy–VMR series report short median hospital stay of about 1 day and low 30-day morbidity and readmission (~2.5%) when logistics and technique are standardized [19].

Mesh-specific complications remain uncommon in pooled series (~1–2%) and appear more sensitive to surgical plane, footprint, suture choice, and meticulous reperitonealization than to mesh category itself [20–22]. Recent reviews show no meaningful difference in recurrence or mesh-related events between biologic and synthetic prostheses [23, 24].

## Where resection rectopexy still fits

When constipation physiology with a redundant sigmoid or proven slow transit is central to the clinical phenotype, RR provides an element that VMR intentionally omits, resection of the lead point in addition to rectal fixation. Observational cohorts and comparative syntheses indicate durable symptom control in carefully selected constipation-dominant profiles, albeit at the expense of anastomosis-related morbidity and longer operative time with higher rates of major complications in pooled analyses [3, 17, 18]. In this context, RR represents a targeted and defensible strategy, whereas in non-slow-transit patients the functional advantages of VMR together with avoidance of an anastomosis generally make VMR the preferable abdominal option.

## A pragmatic agenda

Disagreement across literature reflects measurement more than ideology. Audits and recent bibliometric mapping of the field demonstrate wide heterogeneity in indication, operative steps, and follow-up, and identify VMR as a current research hotspot while also exposing gaps in international collaboration [13]. In line with the two *Controversies* papers, we call for registries with explicit technique descriptors and common follow-up time points. Comparative syntheses already show broadly equivalent aggregate recurrence across abdominal approaches, strengthening the case for prospective, patient-tailored trials rather than omnibus comparisons that dilute signal.

## Conclusions

The optimal approach is to select the procedure that matches the patient’s phenotype and perform it consistently with nerve-sparing technique. Patients should be explicitly counseled on the specific risks, anastomosis-related for RR and, though rare, mesh-related for VMR. Ventral mesh rectopexy is a reasonable preferred abdominal option whenever an anastomosis can be avoided, anterior defects dominate, or combined pelvic-organ prolapse repair is planned, while RR remains valuable when slow-transit constipation or a redundant sigmoid is central. Taken together, the two *Controversies* papers reframe the debate from technique advocacy to phenotype-driven choice, disciplined execution, and standardized outcome reporting—the very agenda that can make today’s disagreements both testable and clinically useful.

## Data Availability

No datasets were generated or analyzed during the current study.
